# Chemical characterization, antioxidant and inhibitory effects of some marine sponges against carbohydrate metabolizing enzymes

**DOI:** 10.1186/2191-2858-2-30

**Published:** 2012-08-16

**Authors:** Mohamed Shaaban, Howaida I Abd-Alla, Amal Z Hassan, Hanan F Aly, Mohamed A Ghani

**Affiliations:** 1 Chemistry of Natural Compounds Department, Division of Pharmaceutical Industries, National Research Centre, Dokki, Giza, 12622, Egypt; 2 Institute of Organic and Biomolecular Chemistry, University of Göttingen, Tammannstraβe 2, Göttingen, D-37077, Germany; 3 Department of Therapeutic Chemistry, National Research Centre, Dokki, Giza, 12622, Egypt; 4 Red Sea Marine Parks, P.O. Box 363, Hurghada, Red Sea, Egypt

**Keywords:** Sponges, Chemical characterization, *α*-amylase, *α*-glucosidase, *β*-galactosidase, Antioxidants

## Abstract

**Background:**

More than 15,000 marine products have been described up to now; Sponges are champion producers, concerning the diversity of products that have been found. Most bioactive compounds from sponges were classified into anti-inflammatory, antitumor, immuno- or neurosurpressive, antiviral, antimalarial, antibiotic, or antifouling. Evaluation of in vitro inhibitory effects of different extracts from four marine sponges versus some antioxidants indices and carbohydrate hydrolyzing enzymes concerned with diabetes mellitus was studied. The chemical characterizations for the extracts of the predominating sponges; SP1 and SP3 were discussed.

**Methods:**

All chemicals served in the biological study were of analytical grade and purchased from Sigma, Merck and Aldrich. All kits were the products of Biosystems (Spain), Sigma Chemical Company (USA), Biodiagnostic (Egypt). Carbohydrate metabolizing enzymes; Î±-amylase, Î±-glucosidase, and Î²-galactosidase (EC3.2.1.1, EC3.2.1.20, and EC3.2.1.23, respectively) were obtained from Sigma Chemical Company (USA).

**Results:**

Four marine sponges; Smenospongia (SP1), Callyspongia (SP2), Niphates (SP3), and Stylissa (SP4), were collected from the Red Sea at Egyptian coasts, and taxonomically characterized. The sponges' extracts exhibited diverse inhibitory effects on oxidative stress indices and carbohydrate hydrolyzing enzymes in linear relationships to some extent with concentration of inhibitors (dose dependant). The extracts of sponges (3, 1, and 2) showed, respectively, potent-reducing power. Purification and Chemical characterization of sponge 1 using NMR and mass spectroscopy, recognized the existence of di-isobutyl phthalate (1), di-n-butyl phthalate (2), linoleic acid (3), *β*-sitosterol (4), and cholesterol (5). Sponge 3 produced bis-[2-ethyl]-hexyl-phthylester (6) and triglyceride fatty acid ester (7).

**Conclusion:**

Marine sponges are promising sources for delivering of bioactive compounds. Four marine sponges, collected from Red Sea at Egyptian coasts, were identified as Smenospongia (SP1), Callyspongia (SP2), Niphates (SP3), and Stylissa (SP4). The results demonstrated that different sponges extracts exhibited inhibitory effects on oxidative stress indices and carbohydrate hydrolyzing enzymes in linear relationships to some extent with concentration of inhibitors (dose dependant). The extracts of sponges (3, 1, and 2) showed, respectively, potent-reducing power. Chemical characterizations of sponges SP1 and SP3 were discussed. Based on this study, marine sponges are considered as talented sources for production of diverse and multiple biologically active compounds.

## Background

Pharmaceutical interest in sponges was aroused in the early 1950s by the discovery of a number of unknown nucleosides: spongothymidine and spongouridine in the marine sponge *Cryptotethia crypta*[1,2]. These nucleosides were the basis for the synthesis of Ara-C, the first marine-derived anticancer agent and the antiviral drug Ara-A [3]. Ara-C is currently used in the routine treatment of patients with leukaemia and lymphoma. More than 15,000 marine products have been described up to now [4,5]; Sponges are champion producers, concerning the diversity of products that have been found [6]. They are responsible for more than 5,300 different products and every year hundreds of new compounds are being discovered [4]. Most bioactive compounds from sponges can be classified as anti-inflammatory, antitumor, immuno- or neurosurpressive, antiviral, antimalarial, antibiotic, or antifouling [5-9].

Exogenous chemical and endogenous metabolic processes in the human body or in the digestive system might produce highly reactive free radicals, especially oxygen-derived radicals, which are capable of oxidizing biomolecules, resulting in cell death and tissue damage. Almost all organisms are well protected against free radical damage by anti-oxidative enzymes such as superoxide dismutase and catalase (CAT), or by chemicals such as carotenoids, polyphenols, and glutathione [10]. However, when the process of antioxidant protection becomes unbalanced, deterioration of physiological functions may occur resulting in diseases and accelerated aging. There is an increasing evidence, indicating that reactive oxygen species and free radical-mediated reactions are involved in degenerative or pathological events such as aging, cancer, coronary heart ailments, and Alzheimer’s diseases [11]. Moreover, the suppression of the oxidative stress and inflammatory were responded through the inhibition of tumor necrosis factor *β*-(TNF-β) signaling [12]. Natural triterpenes isolated from different marine sponges inhibited iNOS expression and the activation of NF-*β*, while polyketides showed antitumoural activity [13]**.** Most screenings of secondary metabolites of biomedical importance from marine sponge extracts reported an inhibitory effects that turned out to be have strongly cytotoxic effects [14,15].

In this article, evaluation of *in vitro* inhibitory effects of different extracts from four marine sponges *Smenospongia* (SP1), *Callyspongia* (SP2), *Niphates* (SP3), and *Stylissa* (SP4) versus some antioxidants indices and carbohydrate hydrolyzing enzymes concerned with diabetes mellitus. The studied sponges were collected from Red Sea, Hurghada, at Egyptian coasts. Alternatively, the chemical characterizations for two extracts of the predominating sponges; SP1 and SP3 were discussed on the bases of different chromatographic and spectroscopic means. In accordance, di-isobutyl phthalate (**1**), di-*n*-butyl phthalate (**2**), linoleic acid (**3**), *β*-sitosterol (**4**), and cholesterol (**5**) were obtained from SP1; while SP3 delivered *bis*-[2-ethyl]-hexyl-phthylester (**6**) and triglyceride fatty acid ester (**7**).

## Methods

Four marine sponges belonging to the genus *Smenospongia* (SP1), *Callyspongia* (SP2)*, Niphates* (SP3) and *Stylissa* (SP4) were collected from Hurghada at El-Gouna and Shaa’b south Giffton island at depth of 5–8 m. Morphologically, the sponges were characterized and specimens of them were deposited at Red Sea Marine parks, P.O. Box 363-Hurgada, Red Sea, Egypt.

The four sponges were individually extracted by DCM–MeOH (2:1), followed by filtration, and the afforded DCM layers were extracted and evaporated *in vacuo* to dryness. Extracts of sponges were applied to a series of chromatographic purifications including Flash chromatography on silica gel (230–400 mesh), Size exclusion chromatography was done on Sephadex LH-20, and PTLC to isolate their produced bioactive compounds in pure forms. Purity of the yielded compounds were monitored by *R*_f_ values were measured on Polygram SIL G/UV_254_ TLC cards. This lead to isolate the following compounds; Linoleic acid; (9Z,12Z)-9, 12-octadecanoic acid (**3**), β-sitosterol (**4**), Cholesterol (**5**), Di-(2-ethylhexyl)phthalate(DEHP)(**6**), and Triglyceride fatty acids mixture (**7**) were assigned with the aid of different spectroscopic means as follows; NMR (^1^ H &^13^ C NMR) was served using Varian Unity 300 (300.145 MHz; and Varian Inova 600 (150.820 MHz) spectrometers. ESI MS (Thermo Finnigan LCQ with quaternary pump Rheos 4000 (Flux Instrument); Thermo Scientific, USA). EI MS(a Finnigan MAT 95 spectrometer (70 eV); Thermo Scientific, USA. GC-MS was measured on a Trace GC-MS Thermo Finnigan chromatograph, ionization mode EI (70 eV).

The *in vitro* antioxidant study of the sponges extracts were carried out using Carbohydrate metabolizing enzymes; α-amylase, α-glucosidase, and *β*-galactosidase. The antioxidant scavenging activity was studied using serial concentrations of different sponge extracts (10:1000 μg/mL) versus DPPH-free radical. The NO-free radical scavenging activity of extracts was determined according to the method of Sreejayan and Rao [16].

## Results and discussion

### Chemical characterization

Extracts of the four marine sponges *Smenospongia* (SP1), *Callyspongia* (SP2), *Niphates* (SP3), and *Stylissa* (SP4) were applied to a series of chromatographic applications, and hence to identify their bioactive constituents chemically using diverse spectroscopic means. Both sponges, SP1 and SP3, were intensively studied. Five compounds were revealed from SP1; di-isobutyl phthalate (**1**), di-*n*-butyl phthalate (**2**), linoleic acid (**3**), *β*-sitosterol (**4**), and cholesterol (**5**). The first two esters, di-isobutyl phthalate (**1**, RT: 13.79, 100%) and di-*n*-butyl phthalate (**2**, RT: 14.84, 12%), were established by GC-MS analysis together with further unknown components of RT 19.03 (17%) and 20.01 (78%). Purification of sponge 3 (SP3) afforded *bis*-[2-ethyl]-hexyl-phthylester (**6**) and triglyceride fatty acid ester (**7**). In contrast, working up and purification of the extracts obtained from the remaining two sponges SP2 and SP4 delivered multi-metabolites, however, with insufficient amounts for analysis.

Based on their chromatographic properties, spectroscopic means (NMR and MS), and comparison with authentic samples and literatures, the obtained structures were deduced as (9Z,12Z)-9,12-octadecanoic acid (**3**) [17], *β*-sitosterol (**4**) [18,19], cholesterol (**5**) [20-24], phthalic acid *bis*-[2-ethyl-hexyl] ester (**6**) [25] and triglyceride fatty acid mixture (**7**) [26].

### Biological study

**Figure 1:**
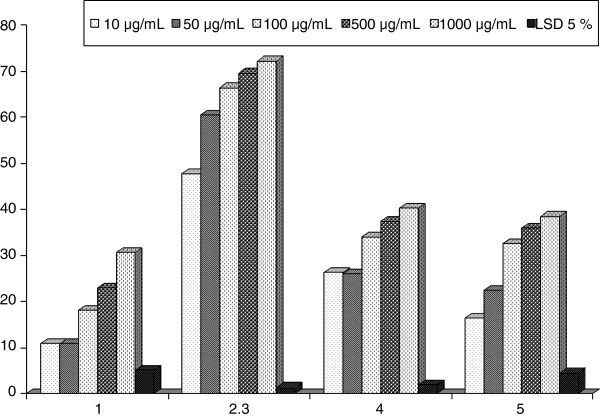
DPPH inhibition percent of the four sponges extracts.

The present results demonstrate the inhibitory effect of different extracts of marine sponges, on antioxidant indices and carbohydrate hydrolyzing enzymes *in vitro*. The 2,2-diphenyl-1-picrylhydrazyl (DPPH)-free radical scavenging effects of different extracts from marine sponge were shown in Table 1 and Figure 1*.* All the tested extracts showed appreciable free radical scavenging activities. Extract of SP2 has the strongest radical scavenging activity at different concentrations compared to other extracts followed by SP3 and SP4. However, SP1 showed the lowest radical scavenging activity. A dose–response relationship was found in the DPPH radical scavenging activity, at where the activity increased as the increase of extracts concentrations. SP2 extract was able to reduce the stable radical DPPH to the yellow color to give significant inhibitory percent 47.7 ± 0.84, 60.53 ± 0.50, 66.45 ± 0.29, 69.52 ± 52, and 72.19 ± 0.69% at concentrations of 10, 50, 100, 500, and 1000 μg/mL, respectively. Alternatively, SP3 showed a reducing inhibitory percent of DPPH amounted 26.31 ± 0.50, 26.00 ± 1.38, 34.04 ± 0.94, 37.39 ± 1.10, and 40.17 ± 0.9% at the same concentrations of extracts, respectively. Moreover, SP4 showed a significant reducing activity of 16.39 ± 4.63, 22.38 ± 1.23, 32.54 ± 1.42, 35.94 ± 1.42, and 38.48 ± 0.70%, respectively. Contrarily, SP1 exhibited the lowest reducing activity compared with aforementioned sponges extracts. The demonstrated inhibitory activity of the DPPH by the sponges extracts might be mainly attributed to their containing of some terpenoidal analogs [26]. 

**Table 1:** DPPH inhibition percent of the four sponges extracts

**Concentrations**	**Extracts of the four sponges**			
	**SP1**	**SP2**	**SP3**	**SP4**
10 μg/mL	10.85 ± 5.42^c^	47.7 ± 0.84^e^	26.31 ± 0.50^d^	16.39 ± 4.63^d^
50 μg/mL	10.77 ± 2.09^c^	60.53 ± 0.50^d^	26.00 ± 1.38^d^	22.38 ± 1.23^c^
100 μg/mL	18.09 ± 1.13^b^	66.45 ± 0.29^c^	34.04 ± 0.94^c^	32.54 ± 2.14^b^
500 μg/mL	22.81 ± 1.81^b^	69.52 ± 0.97^b^	37.39 ± 1.10^b^	35.94 ± 1.42^ab^
1000 μg/mL	30.64 ± 0.60^a^	72.19 ± 0.69^a^	40.17 ± 0.9^a^	38.48 ± 0.70^a^
LSD 5%	5.06	1.26	1.83	4.46

DPPH is expressed %; Data are mean ± SD of 3 replicates; Statistical analysis is carried out using one way analysis of variance (ANOVA) using CoStat: computer program; unshared superscript letters between treatments are significance values at *P* < 0.001.

**Figure 2:**
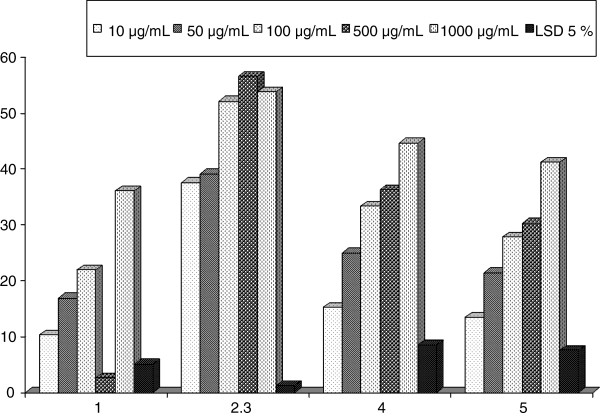
Inhibition percent of NO of the four sponges extracts.

Nitric oxide synthase (NOS) is catalyzing the production of nitric oxide (NO). Inducible NOS (iNOS) is expressed by vascular endothelial cells and smooth muscle cells in response to cytokines, unlike the two other types of NOS, which are constitutive. NO produced by iNOS is implicated in inflammatory diseases [27]**.** NO-free radicals scavenging capacity of the different marine sponges extracts were illustrated in Table 2 and Figure 2. The most reducing capacity was as well considered for SP2, which showed significant ability to reduce the activity of NO by 37.60 ± 1.68, 39.16 ± 9.13, 52.13 ± 6.06, 56.58 ± 3.46, and 53.85 ± 5.12% at concentrations of 10–1000 μg/mL. Alternatively, extract of SP3 exhibited lower significant reducing activity of NO (15.26 ± 6.94, 24.94 ± 2.89, 33.30 ± 2.65, 36.29 ± 5.18, and 44.64 ± 4.29%) with lower extent than those of SP2. Based on the percentage scavenging values, it was remarked that SP1 and SP4 exhibited moderate scavenging effects with linear relationships in a dose-dependent manner. Consequently, extracts of SP1 and SP4 recorded potent reducing capability of 41.24 ± 3.27 and 38.17 ± 3.01%, respectively, at a concentration of 1000 μg/mL. Tasi et al. [28] reported that food and phytochemicals exerts NO-suppressing activity via three different pathways: the blocking of iNOS expression, inactivation of iNOS catalytic function, and the scavenging NO. While NO suppressing effect was primarily through regulation of cellular iNOS expression. The extracts’ effects on the suppressing activity of NO production might be attributed to their containing of polyphenolic compounds or the triterpenes [29]. 

**Table 2:** Inhibition percent of nitric oxide (NO) of the four sponges extracts

**Concentrations**	**Extracts of the four sponges**			
	**SP1**	**SP2**	**SP3**	**SP4**
10 g/mL	10.30 ± 4.29^c^	37.60 ± 1.68^e^	15.26 ± 6.94^d^	13.50 ± 5.53^d^
50 μg/mL	16.92 ± 3.25^c^	39.16 ± 9.31^d^	24.94 ± 2.89^c^	21.41 ± 4.42^c^
100 μg/mL	21.95 ± 2.86^b^	52.13 ± 6.06^c^	33.30 ± 2.65^bc^	27.80 ± 4.32^bc^
500 μg/mL	23.59 ± 1.01^b^	56.58 ± 3.46^b^	36.29 ± 5.18^ab^	30.32 ± 2.74^b^
1000 μg/mL	36.14 ± 2.93^a^	53.85 ± 5.12^a^	44.64 ± 4.29^a^	41.24 ± 3.27^a^
LSD 5%	5.06	1.28	8.49	7.59

NO is expressed as%; data are mean ± SD of 3 replicates; statistical analysis is carried out using one way analysis of variance (ANOVA) using CoStat: computer program; unshared superscript letters between treatments are significance values at *P* < 0.001.

**Figure 3:**
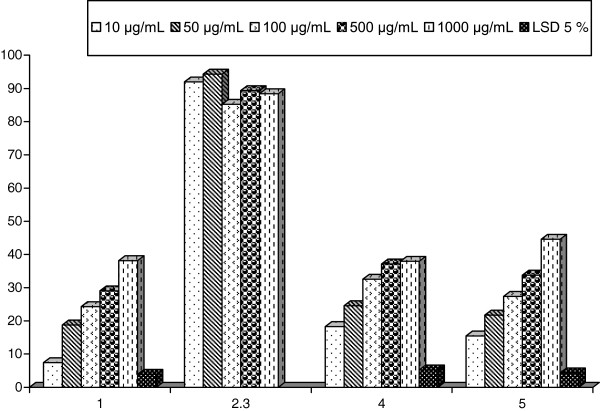
α-Amylase inhibition percent of four sponges extracts.

In alternative manner, extracts of the four sponges were tested against α-amylase carbohydrate hydrolyzing enzyme activity (Table 3, Figure 3). The four sponges showed potent α-amylase inhibitory activity, which may be potentially useful in control of obesity and diabetes. The inhibition of α-amylase by SP3 and SP4 was remarked to be as dose dependent, exhibiting the highest significant reducing activity 44.59 ± 1.55 and 43.64 ± 1.79%, respectively, at a concentration of 1000 μg/mL. Furthermore, extract of SP2 exhibited the most dramatic inhibiting effect at 10 and 50 μg/mL, displaying insignificant reducing activity 92.00 ± 1.21 and 94.35 ± 2.69%, respectively. In addition, the inhibitory activity of SP2 recorded 88.40 ± 7.29% at 1000 μg/mL. Consequently, SP3 recorded a significant inhibitory percent of 18.26 ± 3.97, 24.55 ± 4.03, 32.56 ± 2.07, 37.14 ± 0.89, and 37.97 ± 1.86% in a dose-dependent manner at 10, 50, 500, and 1000 μg/mL, respectively. The anti-amylase inhibitory activity may be due to the ability of phenolic compounds to interact with and/or inhibit proteins enzymes [30]. 

**Table 3:** ***α*-Amylase inhibition percent of four sponges extracts**

**Concentrations**	**Extracts of the four sponges**			
	**SP1**	**SP2**	**SP3**	**SP4**
10 μg/mL	7.41 ± 2.61^e^	92.00 ± 1.21	18.26 ± 3.97^d^	15.44 ± 2.68^e^
50 μg/mL	18.75 ± 2.04^d^	94.35 ± 2.69	24.55 ± 4.03^c^	21.72 ± 3.20^d^
100 μg/mL	24.27 ± 3.07^c^	85.22 ± 4.92	32.56 ± 2.07^b^	27.38 ± 1.73^c^
500 μg/mL	29.05 ± 1.47^b^	89.38 ± 8.22	37.14 ± 0.89^ab^	33.75 ± 2.31^b^
1000 μg/mL	38.13 ± 0.64^a^	88.40 ± 7.29	37.97 ± 1.86^a^	44.59 ± 1.55^a^
LSD 5%	3.9	NS	5.18	4.32

*α*-amylase is expressed as %; data are mean ± SD of 3 replicates; statistical analysis is carried out using one way analysis of variance (ANOVA) using CoStat: computer program; unshared superscript letters between treatments are significance values at *P* < 0.001.

**Figure 4:**
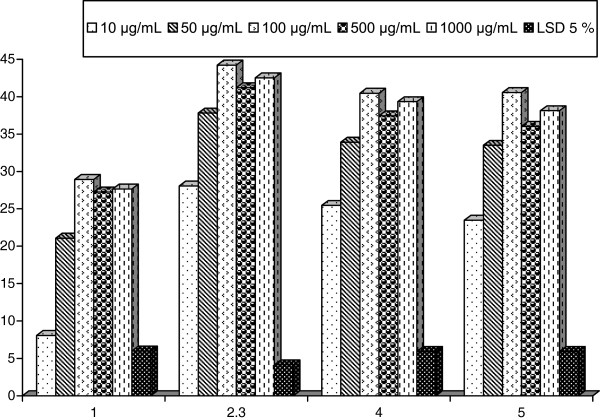
***α*-Glucosidase inhibition percent of the four sponges extracts.**

One therapeutic approach for treating diabetes is to decrease the post-prandial hyperglycemia. This is done by retarding the absorption of glucose through the inhibition of the carbohydrate hydrolyzing enzymes α-amylase, *α*-glucosidase, and *β*-galactosidase in the digestive tract. Inhibition of these enzymes delay carbohydrate digestion and prolong overall carbohydrate digestion time, causing a reduction in the rate of glucose absorption and consequently blunting the post-prandial plasma glucose rise [31]. Many natural resources have been investigated with respect to the antidiabetic and suppression of glucose. The inhibitory effects of the four sponges extracts against α-glucosidase carbohydrate hydrolyzing enzyme activity were further studied as listed in Table 4 and Figure 4. Remarkable greater inhibitory effects of SP2 (40.44 ± 4.34, 39.32 ± 2.60%) and SP3 (40.55 ± 3.08, 38.11 ± 2.70%) than SP1 (32.22 ± 3.96, 29.42 ± 0.62%) and SP4 (28.95 ± 1.44, 27.65 ± 2.37%) were deduced at concentrations of 100 and 1000 μg/mL, respectively. Hence, the inhibition percent was significantly correlated with the increase in concentration of inhibitors. The fact that α-glucosidase and α-amylase showed different inhibition kinetics seemed to be due to structural differences related to the origin of the enzymes [32]. Manosroi et al. [33] attributed the anti-diabetic, anti-inflammatory, anti-tumor, and anti-proliferative effect of many species, to their constituents of mono, sesquiterpenes, phenolic compounds, and flavonoids such as cinnamic acid, caffeic acid, and rosmarinic acid. 

**Table 4:** ***α*-Glucosidase inhibition percent of the four sponges extracts**

**Concentrations**	**Extracts of the four sponges**			
	**SP1**	**SP2**	**SP3**	**SP4**
10 μg/mL	8.06 ± 3.51^c^	28.05 ± 1.63^c^	25.45 ± 4.04^c^	23.47 ± 4.56^c^
50 μg/mL	21.08 ± 4.68^b^	37.80 ± 2.55^b^	33.91 ± 3.15^b^	33.49 ± 1.76^b^
100 μg/mL	28.95 ± 1.44^a^	44.21 ± 2.87^a^	40.44 ± 4.34^a^	40.55 ± 3.08^a^
500 μg/mL	27.24 ± 3.45^a^	41.19 ± 1.59^ab^	37.42 ± 1.14^ab^	36.01 ± 1.78^ab^
1000 μg/mL	27.65 ± 2.37^a^	42.5 ± 2.24^a^	39.32 ± 2.60^ab^	38.11 ± 2.70^ab^
LSD 5%	5.97	4.14	5.93	5.93

*β*-galactosidase is expressed as %; data are mean ± SD of 3 replicates; statistical analysis is carried out using one way analysis of variance (ANOVA) using CoStat computer program; unshared superscript letters between treatments are significance values at *P* < 0.001.

**Figure 5:**
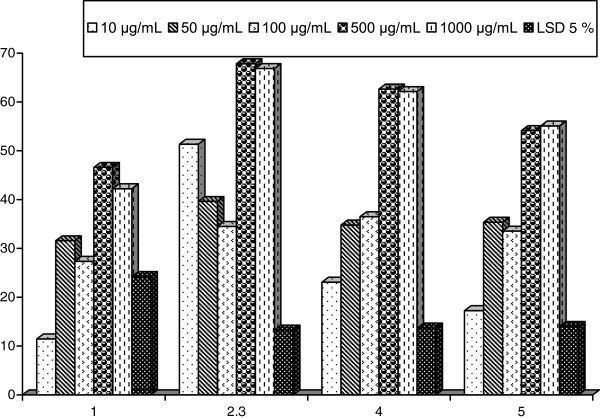
***β*-Galactosidase inhibition percent of four sponges extracts.**

*β*-Galactosidase inhibitory activity was finally studied versus the sponges extracts as summarized in Table 5 and Figure 5. Accordingly, SP2 and SP3 provided additional support for the previous finding by having the strongest reducing activity at various concentrations. Hence, SP2 at 500 and 1000 μg/mL displayed significantly the highest inhibitory percent amounted 67.82 ± 3.94 and 66.86 ± 3.79%, respectively, followed by SP3 (62.63 ± 1.89 and 62.12 ± 4.37%, respectively) and SP4 (54.13 ± 2.44 and 55.08 ± 5.11%, respectively). In contrast, SP1 displayed a comparable insignificant inhibitory activity of 45.89 ± 4.91, 43.77 ± 4.5% at 500 and 1000 μg/mL, respectively. From the manipulated results, it was deduced significant increase in reducing activity with the increase in concentrations of the individual extract (linear relationship).

**Table 5:** ***β*-galactosidase inhibition percent of four sponges extracts**

**Concentrations**	**Extracts of the four sponges**			
	**SP1**	**SP2**	**SP3**	**SP4**
10 μg/mL	11.45 ± 11.54^b^	51.35 ± 5.24^b^	23.09 ± 8.33^b^	17.24 ± 6.39^c^
50 μg/mL	31.58 ± 22.03^ab^	39.62 ± 8.53^bc^	34.78 ± 11.03^b^	35.35 ± 9.72^b^
100 μg/mL	27.37 ± 13.18^ab^	34.49 ± 11.82^c^	36.49 ± 8.35^b^	33.53 ± 11.38^b^
500 μg/mL	46.58 ± 8.22^a^	67.82 ± 3.94^a^	62.63 ± 1.89^a^	54.13 ± 2.44^a^
1000 μg/mL	42.21 ± 5.28^a^	66.86 ± 3.79^a^	62.12 ± 4.37^a^	55.08 ± 5.11^a^
LSD 5%	24.24	13.26	13.7	14.02

*β*-galactosidase is expressed as%; data are mean ± SD of 3 replicates; statistical analysis is carried out using one way analysis of variance (ANOVA) using CoStat computer program; unshared superscript letters between treatments are significance values at *P* < 0.001.

### Experimental

The NMR spectra were measured on Varian Unity 300 (300.145 MHz) and Varian Inova 600 (150.820 MHz) spectrometers. ESI MS was recorded on a Thermo Finnigan LCQ with quaternary pump Rheos 4000 (Flux Instrument); Thermo Scientific, USA). EI mass spectra were recorded on a Finnigan MAT 95 spectrometer (70 eV); Thermo Scientific, USA. GC-MS was measured on a Trace GC-MS Thermo Finnigan chromatograph, ionization mode EI (70 eV), instrument equipped with a capillary column CP-Sil 8 CB for amines (length: 30 m; inside diameter: 0.25 mm; outside diameter: 0.35 mm; film thickness: 0.25 μm); Thermo Scientific., USA. The analysis was carried out at a programmed temperature: initial temperature 40°C (kept for 1 min), then increasing at a rate of 10°C/min and final temperature 280°C (kept for 10 min), injector temperature was 250°C and detector (mode of ionization: EI) temperature at 250°C, He was used as carrier gas at a flow rate of 1 mL/min, total run time 27 min, injection volume 0.2 μL. Flash chromatography was carried out on silica gel (230–400 mesh). *R*_f_ values were measured on Polygram SIL G/UV_254_ TLC cards (Macherey-Nagel GmbH & Co. Germany). Size exclusion chromatography was done on Sephadex LH-20 (Lipophilic Sephadex, Amersham Biosciences Ltd. (purchased from Sigma-Aldrich Chemie, Steinheim, Germany). All chemicals served in the biological study were of analytical grade, which were purchased from Sigma, Merck and Aldrich. All kits were the products of Biosystems (Spain), Sigma Chemical Company (USA), Biodiagnostic (Egypt).

### Sponge materials, collection, and taxonomy

**Figure 6:**
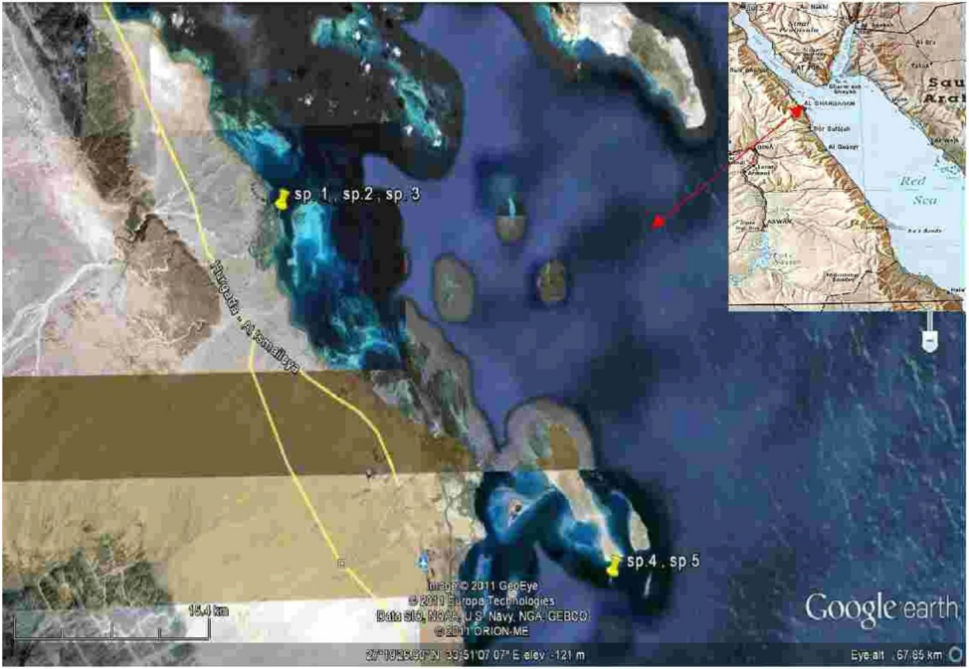
Red Sea satellite map show site locations of the collected sponges.

**Figure 7:**
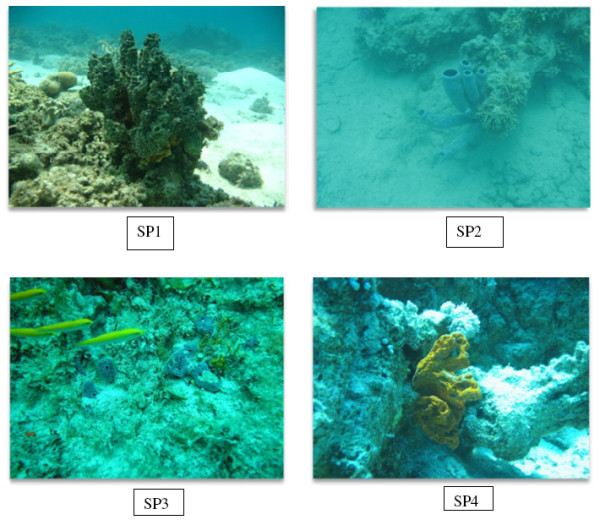
Photos of the collected four sponges (SP1, SP2, SP3, and SP4).

Four varieties of sponges belonging to the genus *Smenospongia* (SP1, olieve, 0.75 kg-wet), *Callyspongia* (SP2*,* faint brown, 0.42 kg-wet), *Niphates* (SP3, faint greenish olieve, 0.15 kg-wet), and *Stylissa* (SP4*,*orange, 0.16 kg-wet) were collected from two sites at Hurghada-coasts, Red Sea, Egypt. The first site located north Hurghada at El-Gouna, latitude N 27° 24′ 06.72″E 33° 41′ 13.52″ and the second site is Shaa’b south Giffton island latitude N 27° 10′ 04.61″ E 33° 57′ 04.87″ (Figure 6). The sponge samples were collected at depth of 5–8 m in September of 2010 (Figure 7) and stored in a freezer until extraction. The four species were morphologically characterized by Mohamed A Ghani, and specimens of them were deposited at Red Sea Marine parks, P.O. Box 363-Hurgada, Red Sea, Egypt.

**Figure 8:**
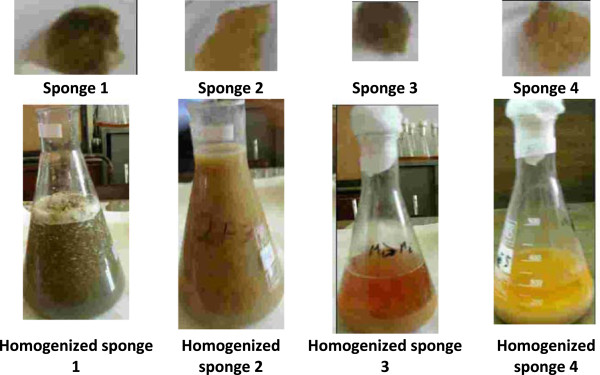
Macerated tissues of the collected four sponges (SP1, SP2, SP3, and SP4).

Taxonomically, the first species (SP1) was belonging to the genus *Smenospongia*, family Thorectidae. Morphologically, the SP1 is Massive with oscular mounds, displaying a bright to dark green coloration. The sponge exuded abundant mucus when handled, exhibiting blunt ends of primary protrude fibers on the surface [34,35]. The second sponge was belonging to the genus *Callyspongia* and family Callyspongiidae [36,37]. Morphologically, it showed bluish to pinkish tubes and sticky massive. Moreover, tissues of the sponge were clear away easily, leaving the clean skeleton. They inhabit as well in the coral reef habitat attached to corals or rocks. The third sponge was belonging to the genus *Niphates* and family Niphatidae. Morphologically, it is massive or encrusting, showing a bluish to purplish grayish cushions and repent branches [36,37]. Finally, the fourth sponge was belonging to the genus *Stylissa* and family Dictyonellidae [36,37]. Morphologically, it is bushy with orange color and tough consistency (Figures 7 and 8). 

### Extraction and isolation

The four sponges, *Smenospongia* (SP1), *Callyspongia* (SP2), *Niphates* (SP3) and *Stylissa* (SP4), were individually cut into small pieces and homogenized mechanically (Figure 1), treated with DCM–MeOH (2:1) and kept at approximately 5°C for 8 days. After filtration, the DCM layers were extracted and evaporated *in vacuo* to dryness affording 1.59, 0.57, 0.39, and 0.64 g from sponges SP1, SP2, SP3, and SP4, respectively. In contrast with sponges SP1 and SP3, both sponges SP2 and SP4 were applied to a series of chromatographic purifications using silica gel, Sephadex, and PTLC affording no desired and inadequate compounds amounts.

#### Working up and purification of smenospongia (SP1)

The afforded greenish-brown crude extract of sponge 1(1.59 g) was subjected to silica gel (column 3 × 60 cm^2^) and eluted with a cyclohexane-hexane/DCM/MeOH gradient. Based on the TLC monitoring, visualized by UV and spraying with anisaldehyde/sulfuric acid, five fractions were obtained: FI (0.1 g), FII (0.2 g), FIII (0.3 g), FIV (0.3 g), and FV (0.4). The fast oil fraction I was applied to GC-MS analysis, displaying a base signal (RT: 25.04 nm, 100%) of unknown component. Fraction II was likely subjected to GC-MS analysis showing four signals representing four components (RT: 13.79, 100%), (14.84, 12%), (19.03, 17%), and (20.01, 78%); the first two of them were of unknown structures, while the last two were assigned as di-isobutyl phthalate (**1**) and di-*n*-butyl phthalate (**2**). TLC monitoring of the remaining fractions (III, IV, and V) recognized their similarity, and they were combined therefore (1.0 g). Consequently, the combined fractions were then chromatographed on silica gel using cyclohexane-DCM–MeOH gradient and after monitoring by TLC, six sub-fractions; PIa (80 mg), PIb (35 mg), PIc (70 mg), PId (95 mg), PIe (38 mg), and PIf (27 mg). An application of the sub-fractions to further purification using Sephadex LH-20 (DCM/MeOH, 60:40) was carried out. In accordance, sub-fractions PIa, PIc afforded a colorless semisolid of linoleic acid (**3**, 55 mg), while purification of sub-fraction PIb afforded a colorless solid of *β*-sitosterol (**4**, 27 mg). Purification of sub-fraction PId afforded a colorless oil of an olefinic fatty acid (23 mg). Similarly, purification sub-fraction PIe yielded a colorless oil of an additional olefinic acid. Finally, an application of the sub-fraction PIf to Sephadex LH-20 (DCM/MeOH, 60:40) resulted in *β*-sitosterol (**4**, 3 mg) and cholesterol (**5**, 3 mg).

#### Linoleic acid; (9Z,12Z)-9, 12-octadecanoic acid (3)

Colorless oil (55 mg) was detected as non-polar UV absorbing band at 254 nm and stained to blue when sprayed by anisaldehyde/sulfuric acid and heated. **–C**_**18**_**H**_**32**_**O**_**2**_ (280). **–*R***_f_ = 0.90 (CHCl_3_/MeOH, 10%). **-**^**1**^ **H NMR** (CDCl_3_, 300 MHz): *δ* = 8.98 (s, br, 1 H, COOH), 5.43–5.28 (m, 4 H, 9,10,12,13-CH), 2.78 (t, ^3^ *J* = 6.0 Hz, 2 H, 11-CH_2_), 2.38 (t, ^3^ *J* = 7.2 Hz, 2 H, 2-CH_2_), 2.08 (m, 4 H, 8,14-CH_2_), 1.63 (m, 2 H, 3-CH_2_), 1.42–1.23 (m, 14 H, 4,5,6,7,16,17-CH_2_), 0.85 (m, 3 H, 18-CH_3_). **–**^**13**^ **C/APT NMR** (CDCl_3_, 50 MHz): *δ* = 180.1 (CO, C_q_), 130.1 (CH-13), 129.9 (CH-9), 128.0 (CH-10), 127.8 (CH-12), 31.5 (CH_2_-2), 29.6 (CH_2_-16), 29.6 (CH_2_-11), 29.5 (CH_2_-14), 29.3 (CH_2_-8), 29.1 (CH_2_-7), 29.0 (CH_2_-6), 29.0 (CH_2_-5), 27.1 (CH_2_-4), 25.6 (CH_2_-3), 24.7 (CH_2_-15), 22.5 (CH_2_-17) 14.0 (CH_2_-18). **–EI MS** (70 eV): *m/z* (%) = 280 (80), 264 (28), 137 (10), 124 (15), 110 (28), 95 (60), 81 (84), 67 (100), 55 (92), 41 (92).

#### β-sitosterol (4)

Colorless solid, UV non-absorbing, turned blue on spraying with anisaldehyde/sulfuric. –**C**_**29**_**H**_**50**_**O** (414). –***R***_**f**_ = 0.51(CH_2_Cl_2_/CH_3_OH 9: 0.5). –^**1**^ **H NMR** (CDCl_3_, 300 MHz): ***δ*** = 5.36 (d, *J* = 4.7 Hz, 1 H, H-6), 3.53 (m, 1 H, H-3), 2.36–2.21 (m, 4 H, H_2_-1, H_2_-4), 2.01–1.93 (m, 2 H, H_2_-6), 1.85–1.75 (m, 4 H), 1.58–1.43 (m, 5 H), 1.21–1.03 (m, 14 H), 0.99 (s, 3 H, CH_3_-19), 0.94 (d, 3 H, *J* = 6.1 Hz, CH_3_-21), 0.86 (d, 3 H, *J* = 6.2 Hz, CH_3_-26), 0.84 (d, 3 H, *J* = 6.2 Hz, CH_3_-27), 0.79 (t, 3 H, *J* = 6.7, CH_3_-29), 0.67 (s, 3 H, CH_3_-18). –**EI-MS** (70 eV): *m/z* (%) = 414 ([M]^+^, 100), 396 ([M-H_2_O]^+^, 37), 381 (21), 329 (34), 303 (41), 283 (16), 259 (10), 241 (22), 227 (9), 206 (11), 189 (18), 173 (22), 151 (13), 135 (25), 123 (21), 109 (18), 83 (13), 43 (15).

#### Cholesterol (5)

Colorless solid, UV non-absorbing, turned blue on spraying with anisaldehyde/sulfuric. –**C**_**27**_**H**_**46**_**O** (386). –***R***_**f**_ = 0.47(CH_2_Cl_2_/CH_3_OH 9:0.5). –^**1**^ **H NMR** (CDCl_3_, 300 MHz): *δ* = 5.37 (d, *J* = 4.7 Hz, 1 H, H-6), 3.51 (m, 1 H, H-3), 2.35–2.19 (m, 4 H, H_2_-1, H_2_-4), 2.02–1.94 (m, 2 H, H_2_-6), 1.85–1.75 (m, 4 H), 1.58–1.43 (m, 6 H), 1.18–1.02 (m, 12 H), 0.99 (s, 3 H, CH_3_-19), 0.94 (d, 3 H, *J* = 6.1 Hz, CH_3_-21), 0.86 (d, 3 H, *J* = 6.2 Hz, CH_3_-26), 0.84 (d, 3 H, *J* = 6.2 Hz, CH_3_-27), 0.67 (s, 3 H, CH_3_-18). –^**13**^ **C NMR** (CDCl_3_, 75 MHz): *δ* = 42.7 (C_q_, C-13), 36.7 (CH, C-1), 140.6 (C_q_, C-5), 56.4 (CH, C-14), 50.2 (CH, C-9), 31.8 (CH, C-8), 56.3 (CH, C-17) 121.8 (CH, C-6), 40.0 (CH_2_, C-24), 32.0 (CH_2_, C-16), 21.0 (CH_2_, C-11), 24.3 (CH_2_, C-15), 37.3 (C_q_, C-10), 28.2 (CH, C-25), 42.3 (C_q_, C-4), 35.9 (CH_2_, C-12), 12.1 (CH_3_, C-18), 31.7 (CH_2_, C-7), 31.2 (CH_2_, C-2), 71.5 (CH, C-3), 19.4 (CH_3_, C-19), 24.0 (CH_2_, C-23), 36.3 (CH_2_, C-22), 18.8 (CH_3_, C-21), 35.8 (CH, C-20), 22.6 (CH_3_, 26), 22.6 (CH_3_, 27). –**EI-MS** (70 eV): *m/z* (%) = 386 ([M]^+^, 100), 368 ([M-H_2_O]^+^, 36), 353 (20), 301 (32), 275 (40), 255 (18), 231 (12), 213 (21), 199 (8), 178 (12), 161 (16), 145 (21), 133 (12), 107 (24), 95 (20), 81 (17), 55 (14), 43 (16).

#### Working up and purification of callyspongia (SP3)

The afforded reddish-brown crude extract of sponge 3 (0.39 g) was subjected to silica gel column (2 × 50 cm) and eluted with a cyclohexane-hexane/DCM/MeOH gradient. According to TLC monitoring, visualized by UV and spraying with anisaldehyde/sulfuric acid, three fractions were obtained: FIa (140 mg), FIb (70 mg), and FIc (60 mg). Purification of FIa using Sephadex LH-20 (DCM/MeOH, 60:40) afforded a colorless oil of phthylester (**6**, 80 mg). Purification of FIb by Sephadex LH-20 (DCM/MeOH, 60:40) resulted in a colorless oil of triglyceride fatty acid ester mixture (**7**, 35 mg). An application of fraction FIc to purification with Sephadex LH-20 (DCM/MeOH, 60:40) afforded a colorless solid of cholesterol (**5**, 28 mg).

#### Di-(2-ethylhexyl)phthalate(DEHP)(6)

UV-absorbing (254 nm) turned intensive violet on spraying with anisaldehyde/sulfuric acid after heating, and changed latter as blue. –**C**_**24**_**H**_**38**_**O**_**4**_ (390). **–*R***_**f**_ = 0.90; CHCl_3_. **–**^**1**^ **H NMR** (CDCl_3_, 300 MHz): *δ* = 7.70 (m, 2 H), 7.50 (m, 2 H), 4.25 (d, ^3^ *J* = 5 Hz, 2 H), 1.80–1.20 (br m, 18 H), 1.00–0.75 (br m, 12 H). –^**13**^ **C NMR** (CDCl_3_, 75 MHz): *δ* = 167.7 (C_q_-1′,1″), 132.4 (C_q_-1,2), 130.9 (CH-3,6), 128.8 (CH-4,5), 68.1 (CH_2_-2′, 2″), 38.7 (CH-3′,3″), 30.3 (CH_2_-4′,4″), 28.9 (CH_2_-5′,5″), 22.9 (CH_2_-6′,6″), 14.0 (CH_3_-7′,7″), 23.7 (CH_2_-8′,8″), 10.9 (CH_3_-9′,9″). –**EI-MS** (70 eV): *m/z*: 390 (3), 279 (20), 149 (64). –**CI-MS** (NH_3_): 798 ([2 M + NH_4_]^+^, 62 %), 408 ([M + NH_4_]^+^, 100), 391 ([M + H]^+^, 65).

#### Triglyceride fatty acids mixture (7)

Colorless oil, turned violet by anisaldehyde/sulfuric acid; –***R***_**f**_ = 0.78 (CH_2_Cl_2_). –^**1**^ **H NMR** (300 MHz, CDCl_3_): *δ* = 5.37 (m, 2 H), 5.34 (m 2 H), 5.27 (m, 1 H), 4.30 (dd, 2 H, *J* = 11.9, 4.3 Hz), 4.14 (dd, 2 H, *J* = 11.9, 6.0 Hz), 2.81 (m, 2 H), 2.31 (m, 2 H), 2.02 (m, 4 H), 1.61 (m, 2 H), 1.40–1.20 (m, 22 H), and 0.88 (t, 9 H, *J* = 6.9 Hz). –^**13**^ **C NMR** (75 MHz, CDCl_3_): *δ* = 172.9 (2C_q_, CO), 172.5 (C_q_, CO), 129.5 (2CH), 129.1 (2CH), 132–127 (further mCH), 68.8 (CH), 62.5 (2CH_2_), 34.0 (CH_2_), 31.9 (CH_2_), 29.8 (CH_2_), 29.7 (CH_2_) 29.6 (CH_2_) 29.5 (CH_2_) 29.4 (CH_2_) 29.3 (CH_2_) 29.2 (CH_2_) 29.0 (CH_2_) 29.1 (CH_2_), 29.0 (CH_2_), 27.2 (CH_2_), 25.6 (CH_2_), 24.9 (CH_2_), 22.7 (CH_2_) and 14.1 (CH_3_).

### *In vitro* antioxidant study

#### Purified enzymes

Carbohydrate metabolizing enzymes; α-amylase, α-glucosidase, and *β*-galactosidase (EC3.2.1.1, EC3.2.1.20, and EC3.2.1.23, respectively) were obtained from Sigma Chemical Company (USA).

#### The antioxidant scavenging activity

The activity of serial concentrations of different sponge extracts (10:1000 μg/mL) on DPPH-free radical will performed according to the method of McCue et al. [36] and Katsube et al. [37]. The decrease in optical density of DPPH^-^ is calculated compared with a control substance as follows:

(1) $$\%\text{Inhibition}=A_{\text{control}}-A_{\text{sample}}\times 100$$

#### Determination of NO-free radical scavenging activity

NO-scavenging activity of extracts was determined according to the method of Sreejayan and Rao [16].

#### Determination of α-amylase

*α*-Amylase was determined according to the method of Bernfeld [38].

#### Determination of β-galactosidase activity

*β*-galactosidase was measured by the method of Sánchez and Hardisson [39].

#### Estimation of α-glucosidase activity

α-glucosidase activity was determined according to the method of Kapustka et al. [40] and Kim et al. [32].

## Conclusions

In conclusion, this study was performed to investigate the effects of the extracts of four marine sponges on some biochemical parameters including antioxidant and three different carbohydrate hydrolyzing enzymes (*α*-amylase, *β*-galactosidase, and *α*-glucosidase). These sponges were collected from Red Sea at Egyptian coasts, which were taxonomically belonged to the genus of *Smenospongia* (SP1), *Callyspongia* (SP2), *Niphates* (SP3), and *Stylissa* (SP4). The results demonstrated that different extracts exhibited inhibitory effects on oxidative stress indices and carbohydrate hydrolyzing enzymes in linear relationships to some extent with concentration of inhibitors (dose dependant). The extracts of sponges (**3**, **1**, and **2**) showed, respectively, potent-reducing power. Chemical characterizations of sponges SP1 and SP3 were discussed, at where di-isobutyl phthalate (**1**), di-*n*-butyl phthalate (**2**), linoleic acid (**3**), *β*-sitosterol (**4**), and cholesterol (**5**) were obtained from sponge SP1; while sponge SP3 produced *bis*-[2-ethyl]-hexyl-phthylester (**6**) and triglyceride fatty acid ester (**7**).

## Competing interests

The authors declare that they have no competing interests.

## Authors' contributions

MS is the principle investigator of the research work, who is responsible for points of research in the manuscript. Additionally, he is the responsible investigator for the structural elucidation of the isolated bioactive compounds, in additions to control the whole manuscript research points. HIA-A and AZH are the chemists who mainly made the main work of extraction and isolation of the bioactive constituents obtained from the sponges under study. HFA is the main investigator who studied the whole biological part of the obtained sponges extracts of the research work under study. MAG is the main investigator who collected the sponges under study together with their full taxonomical study. All authors read and approved the final manuscript.
